# The ex-utero intrapartum treatment (EXIT) strategy for fetal giant sacrococcygeal teratoma with cardiac insufficiency: A case report and review of the literature

**DOI:** 10.3389/fonc.2022.1035058

**Published:** 2022-11-02

**Authors:** Yunping Ding, Mengmeng Yang, Min Lv, Ying Jiang, Tian Dong, Baihui Zhao, Qiong Luo

**Affiliations:** Department of Obstetrics, Women’s Hospital, Zhejiang University School of Medicine, Hangzhou, China

**Keywords:** ex-utero intrapartum treatment (EXIT), sacrococcygeal teratoma (SCT), cardiac insufficiency, fetal tumor, prenatal diagnosis

## Abstract

**Background:**

Antenatally diagnosed sacrococcygeal teratoma has been associated with risks of perinatal complications and death, especially when the foetus has symptoms of cardiac insufficiency, hydrops or anemia *in utero*; however, the method of intervention remains controversial.

**Case:**

A 25-year-old pregnant woman was found to have a cystic and solid tumor in the fetal sacrococcygeal region at 16 weeks of gestation. As the tumour grew, the mother developed polyhydramnios accompanied with gestational diabetes. Fetal and tumorous hemodynamics were closely monitored by ultrasound. Abnormal cardiac function was detected at 31 weeks’ gestation, and we creatively performed pre-emptive delivery through the ex-utero intrapartum treatment with debulking. The teratoma was removed with utero-placental circulation support. The operation proceeded smoothly with favourable prognosis for both mother and newborn.

**Conclusion:**

The ex-utero intrapartum treatment may improve the prognosis for fetuses with heart failure when they reach viable gestation.

## Introduction

Sacrococcygeal teratoma (SCT) is the most common congenital tumour in fetuses and neonates, with an incidence of 1 in 40,000 live births (1). SCT diagnosed postnatally has been associated with an excellent prognosis after surgical excision. In contrast,the mortality excluding terminations in the fetuses with prenatally diagnosed SCT ranges between 35% and 62%, furthermore, for fetuses with tumor volume to fetal weight ratio(TFR) >0.12 prior to 24 weeks gestation, more than 80% of them have poor prognosis (2–4), especially in fetuses with large tumours, rapid growth, and rich blood flow, is still related to a high risk of death.

The two main pathophysiological mechanisms of perinatal death in fetal SCT are the mass effect of the tumour, which can result in preterm delivery or tumor rupture either prior to or at the time of delivery, and the tumour vascular steal phenomenon, which can lead to anaemia and high-output cardiac failure caused by compensatory hypervolemia (5, 6). High-output failure is a more common perinatal complication in clinical practice, which may cause polyhydramnios, hydrops, intrauterine fetal demise and preterm birth.

As a result, to avoid these risks, different surgical approaches have been attempted including open fetal surgery and various minimally invasive therapies, which we will elaborate later in the literature review and discussion. All are aimed at interrupting tumor perfusion, slowing tumor growth and halting or reversing hydrops progression to get good outcomes. If fetal heart failure occurs after viability, early delivery and timely surgery may be the best option to avoid intrauterine death.

The aim of this paper is to demonstrate that a multidisciplinary approach to these tumours with close ultrasound monitoring and timely termination of pregnancy through ex-utero intrapartum treatment(EXIT) may bring positive outcomes for both mother and infant. And also we reviewd the literature regarding therapeutic options in order to assist obstetricians with the reasonable follow-up planning and decision making for achieving a good prognosis for these rare high-risk tumours.

## Case presentation

**Figure 1:**
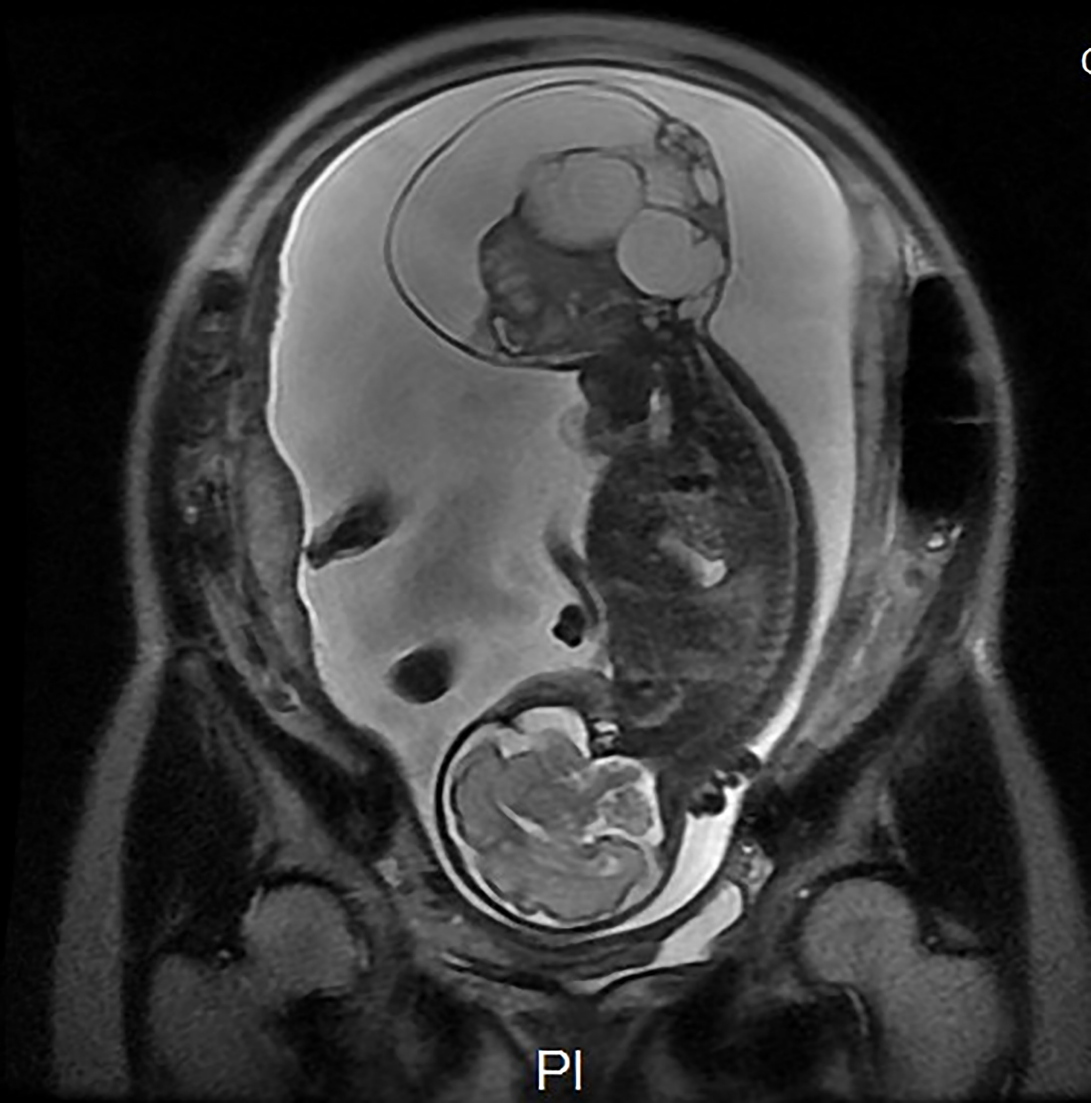
Magnetic resonance imaging was performed at 27 weeks. There was no evidence of possible invasion to the foetal pelvis or abdomen. The intact spine, foetal kidneys, and bladder were normal. A diagnosis of type I in the Altman classification was confirmed. Ax DWI b = 600.

The case was a 25-year-old woman, gravida 2, para 0, with a spontaneous abortion 3 years previously. She had no family history of birth defects or genetic disorders, and she had no alcohol or smoking habits. She had a normal first trimester scan at 12 weeks; however, a sonographic examination revealed a 32 × 22 × 33 mm exophytic, mixed echogenic mass arising from the sacrococcygeal region at 16 weeks. The inner solid size was approximately 23 × 23 × 16 mm, with high vascularization seen on Doppler flow. No other abnormalities were detected, and non-invasive prenatal testing was at low risk. The parents requested to continue the pregnancy after being extensively counselled by our antenatal diagnostic centre regarding the diagnosis, treatment, and prognosis of high-risk SCT. The tumour grew in parallel with the foetus, measuring 107 × 106 × 93 mm and with polyhydramnios (amniotic fluid index 25.4) appearing at 24 weeks. Furthermore, the mother developed gestational diabetes with no other symptoms. Magnetic resonance imaging was performed at 27 weeks (**Figure 1**). There was no evidence of possible invasion to the foetal pelvis or abdomen. The intact spine, foetal kidneys, and bladder were normal. Based on the MRI findings, a diagnosis of type I in the Altman classification was confirmed.

The gradual growth of the SCT was identified by weekly or biweekly sonography, with no signs of foetal cardiac failure until 31 weeks and one day, and the foetal echocardiogram showed that the combined cardiac output increased to 679 ml/kg/min, accompanied by an enlarged cardiothoracic ratio and a full dilation of the inferior vena cava. Multiple large blood vessels arising from the middle sacral artery were supplying the teratoma. Doppler ultrasound, echocardiography and MRI findings are recorded in **Table 1**. Fortunately, the middle cerebral artery-peak systolic velocity(MCA-PSV) was in normal state all the time. Considering the possibility of foetal heart failure, we conducted a multidisciplinary consultation to avoid irreversible complications in succession. Thus, a premature caesarean section and EXIT procedure were scheduled at 32 weeks’ gestation.

**Table 1:** Measurements in the case.

EGA weeks	Mass volume ml	AFIcm	CCO ml/kg/min	cardiothoracic ratio	Tei index of the left ventricle	Tei index of the right ventricle	S/D ratio	Study
16 2/7	11.6	10.3	-	-	-	-	-	US
20 3/7	39.8	15.4	-	-	-	-	3.5	US
24	527.4	25.4	462	0.46	0.32	0.33	2.9	US+echo
26 3/7	792.5	25.1	-	-	-	-	3.2	US
27	824.5	10.9(AFV)	-	-	-	-	-	MR
28 5/7	1288.6	32.2	537	0.45	-	-	2.3	US+echo
31 1/7	1384.8	35.4	679	0.50	0.28	0.22	4.5	US+echo
32	1413.2	33.5	705	0.52	0.38	0.43	4.7	US+echo

EGA, Estimated gestational age; AFI, amniotic fluid index.

CCO, combined cardiac output ; S/D, systolic/diastolic ratio.

AFV, amniotic fluid volume; MR, magnetic resonance.

US, ultrasonography; ech, fetal echocardiography.

**Figure 2:**
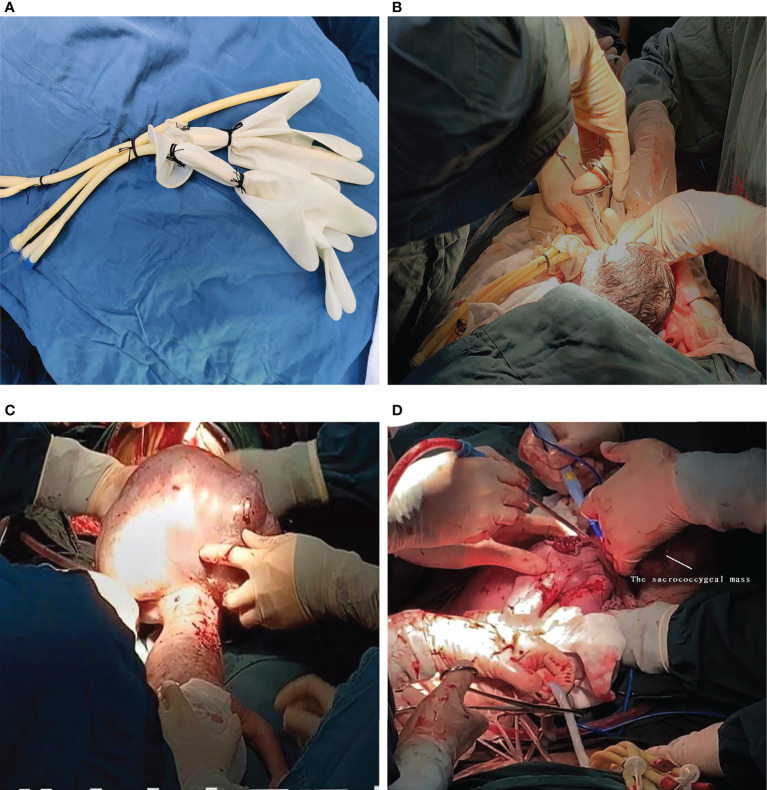
**(A)** The simple homemade balloon. **(B)** Once the foetal head and neck were exposed, the uterine cavity was filled with a homemade balloon continuously infused with warm physiological saline solution. **(C)** The newborn in posterior view with the presence of the sacrococcygeal mass during the EXIT. **(D)** The placental circulation was maintained for a total of 32 min until the intact teratoma was excised without rupture. The umbilical cord was cut immediately upon completion of the tumor debulking procedure.

A multidisciplinary team, including obstetricians, anaesthesiologists, neonatologists, and assistant nurses, was organised for the procedure. After combined epidural and general anesthesia with sevoflurane inhalation was administered as the primary anaesthetic technique, laparotomy was performed with a classic Pfannenstiel approach considering the posterior placenta. The uterine incisions were clamped to reduce bleeding. Once the foetal head and neck were exposed, the uterine cavity was filled continuously with warm physiological saline solution to maintain an adequate uterine volume to avoid placental abruption and compression of the umbilical cord (**Figures 2A, B**). Meanwhile, IV nitroglycerin (NTG) was used to keep the uterus adequately relaxed. Amniotic fluid was slowly released, and the foetus was slowly delivered (**Figure 2C**). The placental circulation was maintained for a total of 32 min until the intact teratoma was excised without rupture (**Figure 2D**). Foetal health signs were stable through SpO_2_ monitoring and foetal ultrasonography during the entire operation. The newborn was then transferred to the neonatal operating table for further suturing and evaluation. The maternal perioperative blood loss was 700 mL, and gauze filled in uterus was removed after 24h to prevent postpartum atony and haemorrhage.

A male infant was born with an SCT of 200 mm in diameter. The weight of the baby was 2200 g, and the teratoma weighed 2065 g. His Apgar score was 8-8. On account of premature delivery, the baby was discharged 19 days after birth with normal MRI results of the abdomen, cerebrum, and kidneys. During the hospital stay, the baby’s condition was stable, and there were no tumour-related complications. Pathological findings revealed an immature teratoma and no malignant elements. Comfortingly, he showed normal development with good heart function and no postoperative complications within 18 months. His mother also did not suffer from postpartum bleeding or infection, and she was discharged without any complications.

## Literature review

With institutional approval, we described our experience with operative treatment in a fetus with large cystic solid SCT and cardiovascular compromise, and reviewed the literature about the perinatal management and postnatal outcomes for fetus with prenatally diagnosed sacrococcygeal teratoma with cardiac insufficiency. We used a list of keywords: (fetal OR prenatal OR antenatal OR fetus) AND (sacrococcygeal teratoma OR sacro-coccygeal teratoma OR SCT) to search the Medline and Cochrane Library computer databases from 2000 to 2020. Only articles in English and cases of neonates or infants with abnormity in hemodynamics were included. The treatment procedure followed ethical principles and approval was obtained from the Institutional Review Board. Written informed consent was obtained from the couple before the procedure and manuscript publication.

There are 6 published articles of SCT with operative treatment for fetal giant sacrococcygeal teratoma with cardiac insufficiency founded in literature search, which are case reports or case series. The characteristics of the cases are summarized in **Table 2** (7–12).

**Table 2:** Overview of literature review of SCT with cardiac insufficiency.

Reference	Cases	Diagnosis GA	Intervention GA	Interventionmethod	Histology	Outcome	Comments
Graf et al. (7)	1	17	23	In utero SCT resection	immature with few malignant yolk sac elements	survived	Cesarean section at 28 weeks’ gestation due to uncontrollable preterm birth
Den Otter (11)	1	27	27	Surgical tumor resection on the second day of life	partly mature and partly immature	survived	Defecation problems developed after 2 months. A second resection of remnants of the SCT took place.
Roybal et al. (8)	8	18-28	26-31	Four underwent SCT resection; One EXIT	two immature; two mature; no data for the rest	five NND; three survived	The only surviving patient delivered before 28 weeks underwent an EXIT procedure.
Goto et al. (9)	1	23	27	Underwent SCT resection	immature	NND	The day after birth the baby suffered non-resuscitable cardiac and pulmonary arrest.
Van Mieghem et al. (10)	5	17-26	17-26	RFA; superficial laser; interstitial laser	two immature; no data for the rest	one NND, two IUFD, two survived	Three cases resulted in preterm labor within 10 days of surgery. One neonate died. Two survived had long-term morbidity related to prematurity.
Baumgarten et al. (12)	11	19-30	27-32	Four underwent debulking and complete resection at 2 months; Five complete resection after delivery;	no data	two NND nine survived	One death was due to severe pulmonary hypoplasia; The second death was due to in utero tumor rup-ture; One patient required additional surgery due to residual tumor, and another required additional surgery for a persistent urogenital sinus.
Present case	1	16	32	EXIT	Immature	survived	He has reached all age-appropriate neurodevelopmental and motor milestones.

GA, gestational age; IUFD, intrauterine fetal death; NND, neonatal death; RFA, radiofrequency ablation.

The series of review comprised 27 fetuses with different degrees of cardiac insufficiency. We reviewed a case of open fetal surgery performed at 23 weeks’ gestation which rarely succeeded after three failed cases (7). Even so, this successful case suffered from premature rupture of membranes and uncontrollable preterm labor at 28 weeks’ gestation. Fortunately, the fetus survived and the routine SCT follow-up was normal. But the majority of other fetuses were not so lucky and ended up with unsatisfactory outcomes due to premature birth, infection, embolism, etc. Therefore, in recent years, new interventions have emerged for giant solid SCT, including ultrasound-guided percutaneous radiofrequency ablation (including vascular ablation and tissue ablation) and laser ablation. The fetal survival rate with minimally invasive treatment was 40% (2/5), but the surviving neonates suffered from long-term complications such as chronic lung disease and developmental delay (10).

In addition, early delivery and immediate resection for fetuses with high-risk SCT have also been proposed (8, 12). In the literature we reviewed, 20 cases underwent premature termination and postnatal tumor resection had a survival rate of 55%(11/20), and 45% (5/11) of the neonates required reoperation due to tumor-related complications (8, 9, 11, 12). An EXIT procedure was performed in only one case at 27 weeks’ gestation, in which the fetus survived, but intraspinal metastasis of the tumor resulted in central nervous system injury (8).

## Discussion

With the improvement of prenatal diagnosis technology, the incidence and detection rate of fetal sacrococcygeal teratoma have increased gradually in recent years (13). Meanwhile, with our further understanding of the disease, people no longer blindly elect termination of pregnancy. However, prenatally diagnosed SCTs are challenging to manage due to their unpredictable nature. No uniform indications for prenatal intervention for SCT have yet been established. According to the summary of previous cases, in cases of singleton, normal fetal karyotype, no other major malformations, evidence of high-output heart failure, gestational age <30 weeks, type I or II, no maternal risk factors for open surgery and contraindications to anesthesia, open surgery may be attempted (14). But there are some common complications such as preterm birth, premature rupture of membranes, and infection after open fetal surgery. Besides, different methods of minimally invasive treatment do not have good outcomes either. In fact, prognosis for these high-risk tumors is uncertain once the fetus has symptoms of heart failure and is in a gestational age that cannot survive after birth, no matter it is open fetal surgery or intrauterine treatment. In order to improve treatment outcome for such patients, it’s not only required to modify intervention measures, but also more about grasping the timing of intervention. Close and dynamic monitoring of fetal hemodynamic changes largely contributed to the success of our case. If there is obvious fetal hydrops or even anemia, surgery may be difficult to reverse the adverse situation.

Although the SCT was detected early in this case, fortunately, the tumor didn’t grow unpredictably rapidly. With our close monitoring, the incipient signs of fetal heart failure were detected in time. As a result, we had to terminate the pregnancy early and rapidly remove the tumour with placental blood flow during delivery, so as to reduce the foetal cardiac load and enable the newborn to establish a normal stable cardiopulmonary circulation system. Theoretically, the foetuses face risk of tumour rupture and bleeding during neonatal transport, EXIT can reduce such risk. In particular, when the foetus has heart failure, it can reduce the incidence of neonatal cardiopulmonary collapse and a series of complications caused by ischemia and hypoxia. EXIT is the most direct and rapid way to break off the blood supply to the tumour and remedy high-output heart failure. During the EXIT, a systematic anaesthetic management can provide profound uterine relaxation, continuous uteroplacental blood flow as well as foetal anaesthesia. Due to the special surgical position of such fetuses, the difficulty of endotracheal intubation increases accordingly, and the implementation of EXIT greatly reduced the possibility of fetal asphyxia caused by failure of intubation. Moreover, the blood supply to the placenta can compensate for foetal blood loss during tumourectomy. For example, one of the high-risk SCT foals studied by Roybal et al (8) underwent the EXIT procedure and survived after surgery. However, the prognosis was poor due to massive invasion of the spinal canal by the tumour. Due to the rarity and unpredictability of SCT, EXIT has rarely been used to treat SCT successfully. Nevertheless, our case report illustrates the successful use of EXIT on fetus with heart failure.

From the above case, it can be concluded that compared with traditional neonatal surgery, EXIT can allow early intervention and safe delivery of patients with SCT. In addition, anaesthesia and foetal ultrasound monitoring during EXIT are conducive to safe and effective operation. The foetal surgical incision is small or does not result in scar formation (15). In comparison with intrauterine surgery, EXIT can avoid the risks such as intrauterine infection, placental abruption, very early premature rupture of membranes and premature delivery. Comfortingly, it can greatly reduce the mental trauma for the family, and its acceptance by patients is higher than that of intrauterine surgery. However, the amount of maternal blood loss during EXIT is higher than that during general caesarean section. MacKenzie et al (16) summarised 31 cases of EXIT and reported that the maternal blood loss was 843 ± 574 mL, which was higher than that during general caesarean section. EXIT may also increase the risk of maternal infection and prolong postpartum recovery time. Hopefully, adverse outcomes will decrease with the application of new surgical instruments and the improvement of techniques (17), such as uterine staplers or modified staplers to avoid the potential of hemorrhage from the relaxed uterus during EXIT and intraoperative uterine packing to prevent postpartum hemorrhage. In addition, the maternal complications associated with EXIT procedures are manageable; indeed, these adverse outcomes had no effect on maternal recovery in our case. Future improvement should be focused on reducing the blood loss associated with the uterine incision, shortening the operation time, and controlling the speed of amniotic fluid loss.

In conclusion, the fetus with giant SCT and heart failure generally has a poor prognosis, so close monitoring by multidiscipline team is critical. When the fetus reaches viable gestation, EXIT procedure would be a good option to improve maternal and perinatal outcomes.

## Data availability statement

The original contributions presented in the study are included in the article/supplementary material. Further inquiries can be directed to the corresponding authors.

## Ethics statement

Written informed consent was obtained from the individual(s), and minor(s)’ legal guardian/next of kin, for the publication of any potentially identifiable images or data included in this article.

## Author contributions

QL, and BZ contributed to the project development and surgical management. ML, TD and YJ were responsible for data acquisition and analysis. YD and MY wrote the manuscript. QL and YD revised the manuscript. All authors read and approved the final version of the manuscript. All authors contributed to the article and approved the submitted version.

## Funding

This study was supported by the center of prenatal diagnosis in Zhejiang province. Funding for this study was provided by the Health Major Science and Technology Project of Zhejiang province grant WKJ-ZJ-2126.

## Acknowledgments

The authors would like to thank Jinhu Wang’s team, doctors from Children’s Hospital Affiliated to Zhejiang University School of Medicine, for their surgery support.

## Conflict of interest

The authors declare that the research was conducted in the absence of any commercial or financial relationships that could be construed as a potential conflict of interest.

## Publisher’s note

All claims expressed in this article are solely those of the authors and do not necessarily represent those of their affiliated organizations, or those of the publisher, the editors and the reviewers. Any product that may be evaluated in this article, or claim that may be made by its manufacturer, is not guaranteed or endorsed by the publisher.
